# Grappling with Proteus: population level approaches to understanding microbial diversity

**DOI:** 10.3389/fmicb.2012.00336

**Published:** 2012-09-11

**Authors:** Mallory J. Choudoir, Ashley N. Campbell, Daniel H. Buckley

**Affiliations:** Department of Crop and Soil Sciences, Cornell UniversityIthaca, NY, USA

**Keywords:** microbial population, ecology, biogeography, evolution, microbial diversity

## Abstract

The emerging fields of microbial population genetics and genomics provide an avenue to study the ecological rules that govern how communities form, function, and evolve. Our struggle to understand the causes and consequences of microbial diversity stems from our inability to define ecologically and evolutionarily meaningful units of diversity. The 16S rRNA-based tools that have been so useful in charting microbial diversity may lack sufficient sensitivity to answer many questions about the ecology and evolution of microbes. Examining genetic diversity with increased resolution is vital to understanding the forces shaping community structure. Population genetic analyses enabled by whole genome sequencing, multilocus sequence analyses, or single-nucleotide polymorphism analyses permit the testing of hypotheses pertaining to the geographic distribution, migration, and habitat preference of specific microbial lineages. Furthermore, these approaches can reveal patterns of gene exchange within and between populations and communities. Tools from microbial population genetics and population genomics can be used to increase the resolution with which we measure microbial diversity, enabling a focus on the scale of genetic diversity at which ecological processes impact evolutionary events. This tighter focus promises to improve our understanding of the causes and consequences of microbial community structure.

## INTRODUCTION

According to Greek mythology, Proteus knew of all things past, present, and future, but only shared this knowledge if captured. When pursued he would change shape, and the closer his pursuer the more quickly he changed, his knowledge just beyond grasp. Our efforts to define the causes and consequences of microbial community structure are like grappling with Proteus. A rigorous framework for understanding microbial community structure remains beyond our grasp because our concepts of what constitutes a community, a species, or a population are ill-developed and fungible. We argue that our comprehension of microbial communities is severely hampered by our inability to recognize ecologically and evolutionarily meaningful units of diversity. We believe observations from microbial population genetics and genomics promise to change the manner in which we define microbial diversity. Population genetics and population genomics both focus on the evolutionary dynamics of populations. While these approaches employ different analytical techniques, conceptually they represent points along a continuum, the differences being the amount and type of genetic information used to make evolutionary inference. Population-based approaches represent a powerful new paradigm for exploring the fundamental units of community structure.

The ribosomal RNA (rRNA) paradigm has been invaluable in charting the diversity of the microbial world by providing both a phylogenetic framework for understanding microbial diversity and tools for characterizing microbial communities without the biases imposed by cultivation. The remarkable conservation of rRNA gene sequences makes them excellent for determining phylogenetic relationships between diverse microbial assemblages. However, as a result of their high conservation these molecules are insensitive to evolutionary changes that occur in response to ecological dynamics. As the field of microbial ecology matures, we increasingly seek to understand the ecological rules that govern how communities assemble, function, and evolve. The rRNA-based tools that have fueled the growth and development of our field may be poorly suited to answering many of the ecological questions we now face.

The most common unit of diversity employed in analyses of microbial community structure is the operational taxonomic unit (OTU) based on the 16S rRNA gene. An OTU is generally defined as a group of sequences that differ by less than 3% of nucleotide positions in the 16S rRNA gene (OTU_0.03_; Hughes etal., 2001). This cutoff is based on current criteria for defining microbial species (Wayne etal., 1987). In other fields of biology, a “species” is considered a distinct and coherent evolutionary unit in terms of ancestry, range, or ecological function (Coyne and Orr, 2004). The current microbial species concept, however, is primarily based on the needs of taxonomic analysis. This taxonomic species concept was established before the use of molecular sequence data and before phylogenetic analysis of microorganisms was possible. Sequence data was applied to the microbial species framework in a *post hoc* manner as a way of supporting the pre-existing species definition. Hence, the foundation that underpins the OTU_0.03_ does not have a firm ecological basis and we should consider carefully the range of ecological and evolutionary hypotheses that can be addressed effectively on the basis of this unit of diversity.

The nascent field of microbial biogeography provides a compelling example of the limitations that the current microbial species concept imposes on our understanding of microbial diversity. As demonstrated by *Escherichia coli* and *Salmonella enterica*, 2.8% divergence of 16S rRNA between species can take approximately 63–120 million years (Ochman etal., 1999). Hence, strains within an OTU_0.03_ may have shared a most recent common ancestor during the early Cretaceous, a period when many of the Earth’s continents were still joined. Consider for a moment the consequences if this unit of diversity were employed in the study of plants and animals. For example, all species in the tortoise family have diverged in the last 50 million years, and thus, if studied using units of diversity that have the same sensitivity as the OTU_0.03_, would represent a single globally distributed taxonomic unit. Were Darwin to have used this definition of diversity, he would have observed only one type of tortoise in the Galapagos, and we would all be the poorer for it. It should not surprise us that this unit of diversity is poorly suited to resolve current patterns of microbial biogeography.

The use of rRNA-based OTUs obscures the recent evolutionary history of microbial lineages. We know that organisms with exactly the same 16S rRNA gene sequence can share as few as 38% of the genes in their genomes (Welch etal., 2002; Tettelin etal., 2005; Hall etal., 2010), and that organisms with the same 16S rRNA gene sequence can have different ecological characteristics (Jaspers and Overmann, 2004). Units of diversity defined by rRNA genes are valuable in terms of discovering and characterizing new lineages, charting the scope of microbial diversity, and resolving evolutionary relationships at temporal scales ranging approximately from 10^7^ to 10^9^ years. However this unit of diversity is not well suited to addressing ecological and evolutionary processes, such as dispersal and speciation, which operate at timescales of less than 10^6^ years.

A focus on genetic diversity at a smaller phylogenetic scale than the OTU_0.03_ is vital to understanding the forces that govern community structure. Rather than attempting to define the appropriate units for studying microbial diversity *a priori*, we should currently be making observations of the patterns of genetic diversity that exist in nature. This can be achieved by focusing on groups of closely related strains and adopting a flexible and methods-free concept of microbial populations: a group of organisms characterized by a genetic, spatial, temporal, or ecological boundary. Populations of isolates can be studied through multilocus analyses which provide greater phylogenetic resolution than 16S rRNA sequence analyses and which allow for estimation of recombination rates. Genomic analyses, including analyses of single-nucleotide polymorphisms (SNPs) or whole genome sequences, provide even greater phylogenetic resolution and the ability to explore patterns of gene exchange and signatures of selection. Population genetics and genomics now provide a solid foundation to study evolutionary dynamics at the sale of ecological interactions and a framework for addressing specific questions such as: (1) How are microbial lineages distributed spatially, and what are the roles of migration and local adaptation in defining the genetic and functional characteristics of communities? (2) How do patterns of gene flow vary with respect to the genetic and geographic distance between strains? (3) How do population dynamics influence community dynamics and ecological processes? The following is a brief overview of how our understanding of microbial ecology may be enhanced by taking a population genomics approach.

## IMPLEMENTING POPULATION-LEVEL APPROACHES

The forces governing microbial biogeography can be best evaluated at fine scales of genetic diversity (Pearson etal., 2009; Vogler etal., 2009), and *Bacillus anthracis* provides a case study to demonstrate this point (Kenefic etal., 2009). The potential use of *Bacillus* *anthracis* in terror attacks created a need to distinguish naturally occurring strains from those used as biological weapons and to understand the genetic diversity within this species. Multiple *Bacillus anthracis* genome sequences were used to identify canonical SNPs that resolve branching points in the phylogeny of the species, and these SNPs were subsequently used to explore the origins of *Bacillus anthracis* in North America (Kenefic etal., 2009). The introduction of anthrax to North America was hypothesized to have occurred along the US Gulf Coast by infected European cattle during the colonial period of American history. However, analysis of SNPs in 285 geographically diverse isolates from North America indicates a Eurasian ancestor originating from the north, likely entering the continent along the Bering land bridge and introduced by ungulate migrations during the last ice age (Kenefic etal., 2009). Through this approach, we see that discernible patterns of microbial biogeography were established as a result of dispersal at a temporal scale of thousands of years. These patterns could only be observed through genome-level analyses.

In another example, Pearson etal. (2009) reconstructed the evolutionary history of *Burkholderia pseudomallei* and *Burkholderia mallei* using > 14,000 orthologous SNPs from 33 whole genome sequences of *Burkholderia pseudomallei* and *Burkholderia mallei* sampled across Australia, Southeast Asia, and the rest of the world. This phylogeny was supplemented with data from multilocus sequence analysis (MLSA) of > 1,700 global *Burkholderia* isolates. Population structure of these isolates supports the existence of two geographically distinct *Burkholderia pseudomallei* subpopulations, originating in Australia and Southeast Asia and separated by the Wallace Line, a geographical pattern well-documented in macroorganisms. The deeply branching Australian *Burkholderia pseudomallei* group was determined to be the most genetically diverse lineage, thus, representative of the ancestral gene pool. Molecular clock estimates suggest these *Burkholderia pseudomallei* populations diverged between 16,000 and 225,000 years ago (Pearson etal., 2009). The biogeographical patterns observed in *Burkholderia* underlie patterns of genome differentiation and ultimately govern the origins of diversity within the genus. These genetic boundaries would be overlooked if sequence analyses were restricted to rRNA genes.

*Helicobacter pylori* inhabits the stomachs of over half the world’s human population and provides another useful case study. MLSA of 769 *H. pylori* isolates representing 51 distinct human populations identified six extant subpopulations of *H. pylori*. These ancestral populations correlate well with geographical regions at a global scale. Extant strains of *H. pylori* demonstrate distinct patterns of ancestral admixture influenced by the geographic origin and mixing of hosts (Linz etal., 2007). There is an inverse relationship between genetic diversity and geographic distance from East Africa in both *H. pylori* and its human host, and thus *H. pylori* dispersal patterns are believed to mirror human migration patterns from East Africa approximately 58,000 years ago (Linz etal., 2007).

These studies demonstrate that patterns of microbial biogeography, veiled in analysis of 16S rRNA genes, become evident through more sensitive genetic analyses. In the case of *Bacillus anthracis*, we learn that European strains did not routinely colonize and persist in North America, despite multiple introductions over hundreds of years during the time of European settlement (Kenefic etal., 2009). This raises interesting new questions about the factors that govern the competitive fitness of strains introduced to new habitats. Issues of dispersal and colonization are critical for understanding constraints on community structure. An advantage of population genetics and genomics is that they provide a route for investigating microbial biogeography and also provide data that can be used to explore the ecological adaptations that impact colonization success and ultimately the environmental distribution of species.

Analyses of strain collections spanning discrete sites make it possible to determine how ecological traits map onto the evolutionary history of a lineage (Connor etal., 2010; Becraft etal., 2011; Preheim etal., 2011). For example, using an approach to map habitat traits onto microbial phylogeny, ecological populations within coastal *Vibrio* isolates can be predicted based on seasonal occurrence and particulate size fractionation (Hunt etal., 2008; Preheim etal., 2011). Populations adapted to a free-living lifestyle can be distinguished from those adapted to living on the surface of organic matter particulates, or on the surface of phytoplankton (Preheim etal., 2011). This approach has also been used to identify ecological populations in *Bacillus* (Connor etal., 2010) and *Synechococcus* (Becraft etal., 2011). Both solar exposure and soil texture are important predictors of ecological populations among the *Bacillus subtilis*–*Bacillus licheniformis* clade (Connor etal., 2010), while ecological populations of *Synechococcus* correspond with gradients of temperature and depth in microbial mats (Becraft etal., 2011). Recognizing the existence of meaningful ecological units is the first step to understanding both the ecological factors that govern the spatial and temporal dynamics of microbial communities and the evolutionary dynamics that govern the origins and maintenance of microbial diversity.

An advantage of using population genomics over single or multilocus methods is the ability to evaluate the impact of horizontal gene transfers (HGTs) on microbial evolution and ecology. HGT can blur the lines of ancestry between lineages, shuffling adaptive genes, and HGT may prevent the development of genetically and ecologically cohesive populations (Fraser etal., 2009; Shapiro etal., 2012). Genomic studies provide evidence that patterns of gene exchange may be controlled by propinquity, with local adaptation facilitated by sampling genes from the environment. For example, environmental co-localization governs exchange of integron cassettes in *Vibrio* species regardless of species boundaries (Boucher etal., 2011), while, interspecies exchange of core genes is not observed (Boucher etal., 2011). Likewise, Caro-Quintero etal. (2011) demonstrated that *Shewanella baltica* isolates in the Baltic Sea exchange genes more frequently with isolates found at similar depths than with isolates at different depths. Lastly, gene flow in the thermoacidophilic crenarchaeon *Sulfolobus islandicus* is influenced by geographical isolation, driving patterns of speciation (Whitaker etal., 2003; Reno etal., 2009). These observations suggest that evolutionary processes may vary between microbial lineages and even between core and auxiliary genes (Riley and Lizotte-Waniewski, 2009; Léfebure et al., 2010). In the case of *Shewanella baltica*, as much as 20% of the genome was inherited from co-localized strains (Caro-Quintero etal., 2011), suggesting that ecological interactions can have strong impacts on genome dynamics.

At a gross level, it is clear that the composition of a microbial community has strong impacts on environmental biogeochemistry, though the biotic and abiotic mechanisms that link community structure and function remain poorly described. If we want to understand community structure–function relationships at a fundamental level, we need to start with measurements of diversity that capture adaptive differences within and between lineages. We need to understand how the genomic diversity within a lineage impacts ecological function and is distributed in the environment, how evolutionary and ecological forces regulate gene exchange, and how patterns of gene exchange within and between lineages impact community function. These inquiries can be enabled by studying populations as the fundamental units from which communities are constructed.

## WHERE DO WE GO FROM HERE?

Microbial population genetics and genomics are opening avenues for understanding the ecological and evolutionary mechanisms governing microbial diversity. However, a focus on the dynamics of populations and species still faces several formidable obstacles. Foremost is the current lack of coherent and pragmatic definitions for populations and species. Without such criteria it is difficult to objectively compare results between studies. As our understanding of microbial populations improves, it will be important to develop objective criteria for defining lineages that will be relevant and applicable across a wide range of microorganisms.

Another challenge to the application of microbial population genomics is a lack of information about how to properly sample the genetic diversity of microbial populations, both in terms of spatial scale and numbers of strains. Rigorous population studies require robust genetic sampling across appropriate spatial, temporal, or habitat scales in order to achieve the ultimate goal of accurately depicting patterns of biodiversity existing in nature. Sufficient individuals must be sampled to represent the breadth of genetic diversity across an organism’s geographic range and to capture genetically informative loci that reflect its evolutionary history. What spatial scales best capture the genomic diversity of microbial populations? Over what spatial and temporal scales does a microbial cell sample genetic material from its environment? What scales are most suitable for inferring adaptive traits from an organism’s habitat distribution? How do we define a microhabitat, or an ecological niche? What environmental parameters should be measured as part of the sampling design? All of these questions are fundamental and must be addressed as we move forward.

An obvious limitation to population genomic approaches is the need for strains to be cultivated in isolation. While most microorganisms have yet to be cultivated, much progress can still be made with organisms that we can cultivate now. Model systems can be developed and used to explore the evolutionary and ecological mechanisms that regulate microbial diversity. Once revealed, these mechanisms can be used to make predictions relevant to organisms more recalcitrant to cultivation. In addition, a solution to the cultivation problem is available through application of single-cell genomics. Single-cell methods of genome analysis can be used to perform genome sequencing or multilocus analysis on individual microbial cells without the need for cultivation (Stepanauskas and Sieracki, 2007; Swan etal., 2011; Tadmor etal., 2011; Martinez-Garcia etal., 2012). Metagenomics offers another avenue through which theory developed through microbial population genetics and genomics may be applied to more complex communities without the need for cultivation (Allen and Banfield, 2005; Simmons etal., 2008; Dick etal., 2009; Morowitz etal., 2011; Denef and Banfield, 2012; Narasingarao etal., 2012).

The ongoing exponential decline in sequencing costs has made population genomics a reality, but the computational tools and theory for understanding these data still lag. While a wide variety of computational population genetics tools exist, many are based on theory developed for eukaryotic organisms. There are fundamental differences between macroorganisms and microorganisms that may impact assumptions implicit in population genetic models. In addition, not all models can equally capture the range of ecological and evolutionary dynamics that operate in the microbial world. For example, the algorithm eBURST determines founding genotypes of clonal complexes from MLSA and has been used to describe evolutionary patterns in many microbial lineages (Feil etal., 2004). However, in populations with high allelic diversity or recombination rates, like *H. pylori*, eBURST may not be appropriate (Turner etal., 2007). The program STRUCTURE uses multilocus data to infer population structure and gene exchange (Pritchard etal., 2000) and has been applied to a number of bacteria including *Moraxella catarrhalis* (Wirth etal., 2007), *H. pylori* (Falush etal., 2003), and *Streptomyces* (Doroghazi and Buckley, 2010). However, it remains challenging to estimate accurately the true number of ancestral populations contributing to a collection of strains. The program LDhat (McVean etal., 2002) is based on coalescent theory and estimates recombination rates from population genetic data. However, LDhat assumes an unstructured population in equilibrium and will misestimate recombination when these assumptions are not met. An alternative to this approach is the program ClonalFrame (Didelot and Falush, 2007), which maps recombination events onto a phylogenetic pattern of clonal ancestry. However, ClonalFrame can have difficulty modeling populations with very high rates of recombination. While each of these tools may be applicable to certain microbial lineages, they can give incorrect results if applied to lineages that violate model assumptions. There is a continuing need to develop computational strategies that focus on microbial population genomics and to test the assumptions that underlie these analyses.

Ultimately, population-level approaches promise to shed light on the forces that govern microbial diversification and evolution. By providing evidence to interpret adaptive traits and identify selective habitats, these approaches will inform our understanding of the competitive interactions within and between lineages, laying the framework for an understanding of community-level interactions. These approaches should also provide insight on the genetic and ecological forces that govern gene exchange. We should focus now on making empirical observations to inform our understanding of the vertical and horizontal components of ancestry and whether and how microbial lineages form ecologically and genetically cohesive units. A better grasp of the relevant units with which to measure microbial diversity is essential for progress in microbial ecology. Insights from microbial population studies promise to improve our understanding of microbial diversity, providing access to knowledge about the causes and consequences of microbial community structure.
